# Sulphide of Calcium as an Antisuppurative

**Published:** 1882-08

**Authors:** 


### Sulphide of Calcium as an Antisuppurative.

Dr. Andrew H. Smith, Chairman of the Committee on Restoratives of the Therapeutical Society of New York, furnishes to the "New York *Medical Journal and Obstetrical Review*" for June, 1882, a report of the committee on the use of sulphide of calcium for the purpose of preventing or diminishing suppuration. After giving the experience of several members of the society, Dr. Smith concludes his report as follows: Judging from this limited number of cases, it would seem that we are warranted in concluding that in many cases of suppurative affections, ranging from the small postules of acne to extensive suppurating surfaces, an appreciable, and often a very marked, benefit is derived from the use of the calcium sulphide, suppuration which would otherwise take place being averted, or the quantity and duration of an existing discharge being lessened. At the same time its action is not uniform; and in many apparently favorable cases it will fail entirely. The drug is somewhat prone to irritate the stomach, and this circumstance affords an indication for small doses frequently repeated instead of larger ones at longer intervals. One-tenth of a grain every two hours in acute cases will generally secure the full therapeutical action of the drug, but larger doses may sometimes be required, and some patients will bear well a grain three or four times a day. Even in small doses the sulphide will occasionally produce headache, and the patient is usually more or less annoyed by eructation of sulphuretted hydrogen.

